# Vagueness and variety in person-centred care

**DOI:** 10.12688/wellcomeopenres.17970.1

**Published:** 2022-06-16

**Authors:** Polly Mitchell, Alan Cribb, Vikki Entwistle

**Affiliations:** 1 Centre for Public Policy Research, King's College London, London, SE19NH, UK; 2 Health Services Research Unit, University of Aberdeen, Aberdeen, AB25 2ZD, UK; 3 Department of Philosophy, University of Aberdeen, Aberdeen, AB24 3DS, UK

**Keywords:** person-centred care, patient-centred care, definition, framework, pluralism, ethics, values

## Abstract

Person-centred care is a cornerstone of contemporary health policy, research and practice. However, many researchers and practitioners worry that it lacks a ‘clear definition and method of measurement,’ and that this creates problems for the implementation of person-centred care and limits understanding of its benefits. In this paper we urge caution about this concern and resist calls for a clear, settled definition and measurement approach. We develop a philosophical and conceptual analysis which is grounded in the body of literature concerning the theory and practice of person-centred care. We consider a range of influential definitional frameworks of person-centred care, highlighting their differences and showing that they do not correspond to a clearly circumscribed and consistent underlying concept. We argue that a degree of indeterminacy and vagueness should not be seen as a problem with the concept of person-centred care; these are features of a rich and contested concept which exists prior to and outside of practical and technical operational definitions and applications. We defend the value of operating with multiple accounts of person-centred care, arguing that what counts as being person-centred can vary across different care contexts, in relation to different patient groups, and as a reflection of different, defensible ethical perspectives. Although the idea of a single, agreed definition is attractive and may seem to be a practical or even necessary step towards meaningful and coordinated action, we argue that this is only the case in a qualified sense. Comprehensive attempts to narrow down the concept in this way should be resisted, as they risk undermining what it is that makes person-centredness a valuable concept in healthcare.

## Introduction

*Person-centred care* and its cousin *patient-centred care* have been high on the healthcare agenda for the past two decades. They were thrust into the limelight by publications such as the Bristol Inquiry into children’s heart surgery deaths, which called for UK National Health Service (NHS) health care to be ‘organised around the patient,’ ^1, 2^ and the Institute for Healthcare Improvement’s manifesto for healthcare in the U.S ‘Crossing the Quality Chasm,’ which identified ‘patient-centredness’—summarised as respect of and responsiveness to patient preferences, needs, and values—as a key dimension of healthcare quality ^3^. Person-centred care is now seen as a cornerstone of health policy and practice internationally. In 2016, the World Health Assembly adopted a Framework on Integrated People-Centred Health Services: services ‘that are organized around the comprehensive needs of people rather than individual diseases’ and which support patients to make decisions about and participate in their own care ^4, 5^. The 2019 Long Term Plan, which set out high-level priorities for England’s NHS for the next ten years, emphasises the need for ‘personalised’ and ‘person-centred’ care, with patients having more choice and control over their care, more individually tailored treatment, and being supported to share responsibility for their health ^6^. In 2022, the Institute for Healthcare Improvement in the U.S stated that ‘[t]he next five years will bring a new era of patient and family engagement’ which puts ‘the patient and the family at the heart of every decision and [empowers] them to be genuine partners in their care.’ ^7^

Despite the significance of person-centred care in contemporary healthcare policy, research, and practice, many researchers and practitioners express worries that it is ‘poorly understood’ or that it lacks a ‘clear definition and method of measurement’ ^8, 9^. This, it is suggested, creates problems for the implementation of person-centred care and limits understanding of its benefits ^9– 13^. However, in this paper we urge caution about such concerns and resist calls for a clear, settled definition and measurement approach. We develop a philosophical and conceptual analysis which is grounded in the body of literature concerning the theory and practice of person-centred care. We argue that a degree of indeterminacy and vagueness should not be seen as a problem with the concept of person-centred care; they are features of a rich and contested concept which exists prior to and outside of practical and technical operational definitions and applications. Person-centred care can, we suggest, mean substantively different things in different care contexts. Although the idea of a single, agreed definition is attractive and may seem to be a practical or even necessary step towards meaningful and coordinated action, we argue that this is only the case in a qualified sense. Comprehensive attempts to narrow down the concept in this way should be resisted, as they risk undermining what it is that makes person-centredness such a valuable concept in healthcare: firstly, its responsiveness and adaptability to the needs of different people and the demands of different contexts; secondly, its full potential to challenge biomedical norms when they become problematic and to draw a wider range of human values and social purposes into the frame of healthcare.

The paper begins with a descriptive and analytical emphasis but as it proceeds we also develop a positive argument. In section two, we outline the concept of person-centred care and give a brief overview of its history and application. In section three, we review the multidimensionality of person-centred care, and identify the range of aspects that healthcare researchers and practitioners have distinguished. We show how different elements of person-centred care are emphasised by different people and institutions and we argue that the different dimensions identified do not correspond to clearly distinct concepts. In section four, we argue that the level of indeterminacy this produces should not be seen as a sign of something problematic. We suggest that value can be attached to vagueness alongside the value that can be attached to definition. In section five, we defend the value of operating with multiple accounts of person-centred care on the grounds that what counts as being person-centred can vary across different care contexts, in relation to different patient groups, and as a reflection of different ethical perspectives. In section six, we conclude that efforts should be made to keep the definition and measurement of person-centred care open and plural. We suggest that of over-riding importance in understanding person-centred care is that the meaning of person-centredness is responsive to particular circumstances. While this will inevitably limit the generalisability of claims that can be made about person-centred care, it is necessary to ensure that it does not become a platitudinous buzzword.

## Person-centred care

A great deal of ink has been spilt over person-centred care; a bewildering array of definitions, interpretations, and operationalised accounts exist. And it is no small irony that many researchers lamenting the absence of a clear definition themselves offer new models and definitions of person-centred care, thus adding to the already-crowded conceptual space. This paper offers an analysis of person-centred care which aims to reflect on and work with the range of existing conceptual and applied research rather than add in another substantive definition of the concept. Although we avoid offering our own detailed representation of the object of inquiry, we recognise the need to say something about what person-centred care is. We therefore begin by drawing on existing literature, seeking to situate ourselves within a practical and theoretical landscape without significantly closing down the definition and scope of person-centred care.

Very generally, the ideas of person-centred care and person-centredness are used to describe healthcare practice that recognises, emphasises, and treats the patient as a *person*—someone for whom health and disease is only part of a fuller and richer life and who has a range of commitments, values, preferences, and experiences as well as skills, knowledge, and capabilities that are relevant to their safe, effective, and appropriate care and treatment ^14^. As such, communication and the relationships between a patient and their healthcare professionals are central to person-centred care, both in order to express this recognition and also as a means of eliciting and better understanding each patient’s perspective. Person-centred approaches often characterise patients as ‘experts’ about their personal and family history, socio-economic circumstances, symptoms, and disease experience ^15, 16^. Positioning a patient as an expert situates them as in some respect equal to their healthcare professionals—with good medical decision-making dependent on each independently bringing relevant knowledge and experience to the clinical encounter. Person-centred healthcare encounters are thus seen as sites of reciprocal information exchange and shared decision-making.

The next section explores definitions and dimensions of person-centred care in more detail. But first we offer some brief contextual remarks on the history of person-centred care; its relationship to more traditional biomedical approaches to healthcare; the perceived value of and reasons for endorsing person-centred care; and finally the terminology used in this conceptual space.

Though they have acquired greater recognition over the past two decades, *person-centredness* and *patient-centredness* have been used to pick out and describe a characteristic and an aim of healthcare for well over fifty years, with roots in humanist psychology and psychoanalysis. In her 1969 address to the UK Royal College of General Practitioners, psychoanalyst Enid Balint spoke of a new approach to medical thinking—patient-centred medicine—in which doctors try to understand their patients’ illness within the context of the whole person, the ‘unique human being,’ before them ^17^. Balint’s husband, Michael Balint, had previously, in his 1964 book *The Doctor, his Patient and the Illness*, emphasised the therapeutic importance of communication and the physician-patient relationship for general practitioners, drawing on the tools and concepts of psychotherapy ^18^. Earlier still, psychologist Carl Rogers pioneered a ‘person-centred approach’ which saw therapy as a way of helping people to connect with their values, self-actualise, and realise their full potential, requiring therapists to see and value their clients in all aspects of their humanity ^19^. The emergence of person-centred care out of psychotherapeutic practice and its initial implementation in general practice contexts may partly explain why it is commonly invoked in primary care and long-term care contexts, including in relation to dementia and mental-health care. The relative importance of communication and relationships between patients and healthcare professionals in these care contexts is also crucial in understanding the uptake and endorsement of person-centredness in these domains ^20^. But the value of person-centred care is increasingly being seen in acute healthcare contexts too ^16^.

Person-centred care is perhaps best understood in relation to what it is not. It is often defined in contrast to or as representing a rejection of a ‘biomedical approach’ to care, which is focussed on diagnosing and treating or curing symptoms and diseases—where these are understood to be faults in a body or in bodily function ^1^ ^8– 10, 17, 21– 24^. Biomedical approaches can be understood in various ways, but broadly they understand health in physical and biochemical terms ^26^ and see healthcare professionals and institutions as responsible for delivering healthcare, while patients are its recipients and beneficiaries ^8, 9^. By contrast, person-centred approaches see health as a ‘biopsychosocial’ phenomenon—with health and disease caused and shaped by psychological and social factors as well as biological ones ^9^. In this sense, person-centredness is closely related to public health and health promotion, which also emphasise the role of social, cultural and psychological factors in health, disease, and health-related risk factors ^27^. Whereas biomedical approaches emphasise evidence-based medicine, seeking to ground good clinical care and decision-making in empirical research evidence and especially population-level experimental data, advocates of person-centred care place emphasis on the context-specific requirements of medicine ^15, 28^. That is, person-centred care requires healthcare professionals to first understand the needs, values, circumstances, and capacity of the person sitting in front of them in their office, before making any assessment about clinically appropriate courses of action. Though it is not always made clear in the literature, biomedical and biopsychosocial approaches are not mutually exclusive. Biomedical approaches can recognise the psychosocial elements of health and disease; biopsychosocial approaches see people in biological and biomedical terms—though not exhaustively so—and can apply generalisable clinical standards and population-level research findings in decision-making at the level of individual patients. The two perspectives reflect different emphases, which can inform, for example, an understanding of the primary functions of healthcare in institutions or healthcare improvement priorities.

Despite this distancing from biomedical healthcare, one reason that person-centred approaches to care are recommended is that they can help to secure biomedical objectives: person-centredness increases the likelihood—in principle at least—of good, sustainable clinically-defined outcomes, compared to traditional biomedical approaches. Understanding a patient’s personal history and social context can expose the social and biological aetiology of their disease, which can be necessary both for accurate and complete diagnosis and identifying appropriate options for treatment and management. This is likely to be particularly salient in the case of complex diseases with both genetic and environmental factors, such as asthma, obesity, and depression, as well as diseases which have variable clinical expression and individual experience ^29^. There are, moreover, biomedically instrumental reasons for foregrounding the patient as a person with personal values, skills, knowledge, interests, and priorities. Knowledge of a patients’ values, circumstances, and capacities can indicate when certain treatment and management strategies are less likely to be appropriate or effective in practice and whether patients are likely to want to and to be able to adhere to them. With respect to long-term conditions, most care and disease management is done by patients and family members rather than doctors and achieving good biomedical outcomes depends on patients taking actions such as following a medication regimen, adopting lifestyle changes, and monitoring changes in their symptoms. This requires patients to have a certain degree of knowledge about their disease and its management, as well as motivation to adopt clinically-indicated behaviours and a willingness to prioritise health amongst other commitments. Notwithstanding biomedical outcomes, person-centred care is valued because it can lead to improved quality of life, patient satisfaction and experiences of care; ends which are increasingly seen as crucial objectives of healthcare services in themselves. These more subjective and experiential outcomes can be in tension and even conflict with biomedical outcomes—improving a patient’s quality of life and giving them a good experience of care can involve deviation from standard care pathways and clinical guidelines ^15^.

The instrumental value of person-centred care for biomedical outcomes and patient satisfaction is to some extent substantiated in the literature. While the relationship between person-centred care and outcomes is decidedly mixed overall, there is some evidence of positive association with biomedical markers and some long-term clinically-defined outcomes and stronger evidence of a relationship between person-centred care and patient satisfaction and quality of life—which may in turn support adherence and self-management behaviours ^30, 31^. Aspects of person-centred care have been shown to produce tangible benefits—improved communication can lead to increased satisfaction, quality of life and better biomedical outcomes ^10, 21, 32, 33^, involving patients in partnership has the potential to reduce adverse outcomes connected to prescribing and healthcare errors ^34, 35^, and patients' perception of the patient-centredness of care is associated with fewer diagnostic tests and referrals ^8^. It is important to note that generating evidence to make causal claims about person-centred care requires person-centred care to be operationalised as an independent variable (or set of independent variables), in order that its effects can be assessed. As we go on to discuss, such operationalisation is contested and not at all straightforward.

Alongside its instrumental value, person-centred care is also valued, not as a means to other valued ends like biomedical outcomes, well-being, or satisfaction but for its own sake, irrespective of its outcomes. The practice of person-centred care is endorsed as treating patients with respect, dignity, and compassion, involving them and recognising them as equals, and as affirming and empowering them ^9, 10, 16, 32, 36– 40^. The non-instrumental value of person-centred care underscores the distinctively ethical complexion of treating someone as a *person*, which accords them a distinctive status and demands and prohibits certain ways of relating to them—requiring, for example, respect for their autonomy, recognition of their capacity to suffer, calling them by their name, and not treating their body as a mere object. This suggests a more fundamental divergence from biomedical approaches: a requirement that the practice of medicine and the design of healthcare institutions reflect broader social values, including some core values which do not straightforwardly concern biomedical success, and which may sometimes be in tension with it. In this section we have relied upon a very general and vague account which essentially contrasts healthcare oriented towards ‘ *persons*’ with healthcare that is more narrowly conceived. Later in the article, we will return to this account as part of a defence of the value of vagueness.

We conclude this section with some remarks about terminology. We have been using the term ‘person-centred,’ but this sits alongside and overlaps with other terms, including ‘patient-centred,’ ‘people-centred,’ and ‘personalised.’ We prefer ‘person-centred,’ which we take to be the most general of this cluster of terms, but we recognise the value of other terms. The terminology ‘person-centred’ helpfully reflects that the approaches and characterisation in question typically indicate a departure from healthcare that is focussed on illness and disease, or health technologies and therapies, and instead try to see patients as whole people who exist in a rich social and personal context and have complex and diverse interests and values, including those associated with their agency and emotional well-being. While many people use ‘patient-centred’ almost interchangeably with ‘person-centred,’ we suggest that the latter more readily transcends institutional categories and boundaries; ‘patient-centred,’ conversely, is situated squarely within a healthcare space ^8^. ‘Person-centred’ also enables the recognition that doctors, nurses, family-members, and anyone else involved in or affected by healthcare are ‘persons’ too, and that their needs and perspective must also be considered ^41^.

Our use of ‘person-centred’ over other designators does not reflect a desire to promote this term exclusively. Other words and phrases in this conceptual space can be used to pick out and emphasise aspects of person-centred care and may be more appropriate than ‘person-centred’ in certain contexts. For example, ‘patient-centred’ can be usefully invoked in order to emphasise the perspective of patients in contrast to doctors, healthcare professionals, healthcare institutions, or other actors who have traditionally had the balance of power in their favour in healthcare contexts. ‘People-centred,’ the phrase preferred by the World Health Organization (WHO), reflects an emphasis on individuals being situated within communities and the need for healthcare services and practices to reflect community values ^5^. This emphasis will be particularly important when thinking about the ways that healthcare institutions and systematically overlook the needs and interests of certain vulnerable populations.

## Multidimensional frameworks of person-centred care

Person-centred care is widely recognised to have multiple dimensions. There have been a number of attempts to develop systematic frameworks of person-centred care, for example via literature review ^9, 10, 42^, empirical study of the views of patients and clinicians ^33, 43, 44^, and conceptual analysis ^15, 45– 47^. All of these identify multiple dimensions, and each has a slightly different focus. Other scholars and practitioners develop or use multidimensional conceptions of person-centred care which adapt and combine existing frameworks and draw on professional experience ^21– 24, 36^. In this section, we set out the variety of dimensions of person-centredness that have been proposed; our catalogue is not exhaustive, but it is relatively extensive. In identifying and fleshing out some of the core dimensions of person-centredness, we show that the ways that different definitions and frameworks characterise and divide up the conceptual space are not consistent and, moreover, there are not clean joints between the different dimensions of person-centredness, which have a tendency to smudge together.

A multidimensional concept has multiple, irreducible parts. This means that it is constituted by several distinguishable components, which make distinctive contributions to the overall concept, and which cannot be fully explained in terms of or subordinated to one another. The dimensions of a multidimensional concept can inform one another and interrelate, but each makes a discrete contribution to the concept. The relationship between the dimensions of a multidimensional concept and the overarching concept is *constitutive*. This means that person-centred care is not separable from its dimensions—for healthcare to be characterised by the dimensions of person-centred care *just is* for it to be person-centred care. ^2^

Our selection here includes seminal multidimensional definitions and frameworks of person-centred care which have influenced other researchers and practitioners ^10, 15, 43– 46, 48^, as well as definitions adopted by institutions which influence policy and practice ^3, 49, 50^. Table 1 sets out these key definitions and frameworks. We supplement our discussion with reference to others who build on, engage with, and use these frameworks.

**Table 1.** Dimensions of person-centred care according to key definitions and frameworks.

Reference	Multidimensional definition
Gerteis *et al.*, 1993 ^43^ (Institute of Medicine, 2001 ^3^ adopts the first six dimensions in their own definition of patient-centred care)	Seven dimensions of person-centred care: 1) respect for patients’ values, preferences and expressed needs; 2) co-ordination and integration of care; 3) information, communication, and education; 4) physical comfort; 5) emotional support and alleviation of fear and anxiety; 6) involvement of family and friends; 7) transition and continuity.
Stewart *et al.*, 1995 ^48^	Patient-centred care… 1) explores the patients' main reason for the visit, concerns, and need for information; 2) seeks an integrated understanding of the patients' world—that is, their whole person, emotional needs, and life issues; 3) finds common ground on what the problem is and mutually agrees on management; 4) enhances prevention and health promotion; 5) enhances the continuing relationship between the patient and the doctor; 6) is realistic given the time and resources available.
Stewart *et al.*, 2013 ^44^ (updated version of model from Stewart *et al.*, 1995 ^48^)	Four Interactive Components of the Patient-Centered Clinical Method: 1) Exploring Health, Disease, and the Illness Experience; 2) Understanding the Whole Person; 3) Finding Common Ground; 4) Enhancing the Patient-Clinician Relationship.
Mead and Bower, 2000 ^10^	Five ways in which person-centred medicine differs from the biomedical model: 1) taking a biopsychosocial perspective; 2) seeing the patient as a person; 3) sharing power and responsibility; 4) working to maintain the therapeutic relationship or alliance; 5) acknowledging the doctor-as-person.
McCormack and McCance, 2006 ^46^	Five person-centred processes: 1) working with patient’s beliefs and values; 2) engagement; 3) having sympathetic presence; 4) sharing decision-making; 5) providing for physical needs; underpinned by prerequisite attributes of nurses: 1) being professionally competent; 2) having developed interpersonal skills; 3) being committed to the job; 4) being able to demonstrate clarity of beliefs and values; 5) knowing self; in a care environment which is characterised by: 1) an appropriate skill mix; 2) systems that facilitate shared decision-making; 3) effective staff relationships; 4) supportive organizational systems; 5) the sharing of power; 6) the potential for innovation and risk-taking; and with a range of expected outcomes: 1) satisfaction with care; 2) involvement with care; 3) feeling of well-being; 4) creating a therapeutic culture.
Leplege *et al.*, 2007 ^15^	Person-centredness means: 1) addressing the person’s specific and holistic properties; 2) addressing the person’s difficulties in everyday life; 3) the person as an expert: Participation and empowerment; 4) respect the person ‘behind’ the impairment or the disease.
The Health Foundation, 2015 ^49^	Four principles of person-centred care: 1) Affording people dignity, compassion and respect; 2) Offering coordinated care, support or treatment; 3) Offering personalised care, support or treatment; 4) Supporting people to recognise and develop their own strengths and abilities to enable them to live an independent and fulfilling life.
WHO, 2016 ^50^	People-centred care… 1) consciously adopts the perspectives of individuals, families and communities; 2) sees individuals, families and communities as participants as well as beneficiaries of trusted health systems that respond to their needs and preferences in humane and holistic ways; 3) requires that people have the education and support they need to make decisions and participate in their own care; 4) is organized around the health needs and expectations of people rather than diseases.

While there are themes that resonate across this set of frameworks, it is striking how diverse they are. This diversity manifests itself both in the dimensions included and, where these are shared across frameworks, the way these are expressed and emphasised. In part, this variety reflects a diversity of purposes—some of the frameworks are developed for use in specific care contexts or in relation to specific healthcare professionals. But there remain a number of ambiguities, of which we highlight four. First, the scope of person-centred care is unclear. Second, the characterisation of specific dimensions of person-centred care is ambiguous. Third, the distinctions and boundaries between different dimensions of person-centred care are uncertain. Fourth, it is unclear whether person-centred care is best understood to describe a process or the end state of a process. We unpack each of these in turn.

### i.  Scope of person-centred care

The scope of person-centred care—what is included and what is excluded—is differently conceptualised across these frameworks. Various dimensions are included in some, but not all, definitions. Only some frameworks emphasise, for example, physical comfort and responding to physical needs ^3, 9, 43, 46^, coordination and continuity of care ^3, 9, 37, 42, 43, 49^, and the role of family, friends and community ^3, 9, 43, 50^. The relationship between doctor (or healthcare professional) and patient and the value of communication seem to be less central in some cases ^49, 50^. The need to be realistic about the available time and resources is only highlighted in the framework developed by Stewart *et al*., along with others using that framework ^33, 48^, and the role of supportive institutions and an adequate care environment is rarely explicitly emphasised ^42, 46^. In their updated version of the model, Stewart *et al*., remove the ‘being realistic’ component, saying that this is ‘not so much a component as a comment on the context within which the patient-centered clinical method is enacted ^44^.’ This highlights that the absence of a dimension in a multi-dimensional person-centred care framework is itself ambiguous: it could indicate that it is not considered to be part of or relevant to person-centred care, that it is not considered to be a *core* part of it, or that it is part of a related but distinct process or phenomenon.

### ii.  Characterisation of dimensions

Where similarly labelled dimensions feature in several frameworks, the specific characterisation of them is not necessarily also shared. Some of the key themes which are repeated across the definitions include recognition of a patient’s values, preferences and concerns; seeing the patient as a person with needs and interests which extend beyond their immediate health problems; and finding common ground and sharing decision-making. However, these are differently conceptualised across definitions: Mead and Bower distinguish between seeing the patient as a person and adopting a biopsychosocial approach ^10^, and Leplege *et al*., similarly distinguish between ‘addressing the person’s specific and holistic properties’ and ‘[respecting] the person ‘behind’ the impairment or the disease ^15^.’ Stewart *et al*., by comparison, combine both of these in a single dimension, that is, seeking an integrated understanding of a patient’s world - their whole person, emotional needs, and other life issues, including their broader social context ^44, 48^. For McCormack and McCance, seeing the patient as a person does not explicitly appear at all in their definition, although they do emphasise the need for nurses to engage with the physical and emotional needs of patients as well as their beliefs and values across their key person-centred processes ^46^. While all the key definitions in Table 1 recognise sharing decision-making and working in partnership as central to person-centred care, they emphasise this differently—the Health Foundation ^49^ and the WHO ^50^ both highlight the need to support patients to develop the knowledge and abilities needed to manage their care independently ^49, 50^, whereas others focus more on building mutual agreement and sharing decision-making between patients and healthcare professionals ^44, 46, 48^. Gerteis *et al*., and the Institute of Medicine place least emphasis on sharing decision-making, instead including the less obviously reciprocal dimension ‘information, communication, and education ^3, 43^.’

This vagueness in the specific meaning of dimensions is perhaps unsurprising—the dimensions of person-centred care do not typically pick out specific and easily identifiable attributes and behaviours. Components such as ‘seeing the patient as a person’ or ‘enhancing the clinician-patient relationship’ are themselves complex and normative phenomena.

### iii.  Boundaries between dimensions

As well as ambiguity about the conceptual scope of person-centred care as a whole, there is linked ambiguity about its internal boundaries. Looking across different definitions of person-centred care—and especially when also considering the ways that other researchers and practitioners have used, developed and built on the key frameworks—we find it difficult to maintain a sense of several clearly distinct and stable dimensions. Rather, what may seem like relatively clear distinctions between dimensions within particular frameworks start to feel less clear-cut when seen in the context of other frameworks. For example, consider the dimension broadly captured by the idea of seeing and treating the patient as a person. This is characterised in a variety of ways:

- Seeing the patient as a person ^10^
- Organized around the health needs and expectations of people rather than diseases ^50^
- Treating people as individuals ^22^
- Seeks an integrated understanding of the patients' world—their whole person, emotional needs, and life issues ^44^
- Understanding the whole person: the person (e.g., life history, personal and developmental issues); the proximal context (e.g., family, employment, social support); the distal context (e.g., culture, community, ecosystem) ^48^
- Understands the whole person: personal and developmental issues (for example, feeling emotionally understood) and the context (the family and how life has been affected) ^33^
- Respect the person ‘behind’ the impairment or the disease ^15^
- Addressing a patient’s physical and emotional needs ^42^
- Respect for patients’ values, preferences and expressed needs ^3, 43^
- Patient participating as a respected and autonomous individual ^42^

These characterisations broadly, though imprecisely, cover very similar conceptual ground. But they reach out in different directions and signal towards other dimensions—some are close to dimensions such as ‘affording people dignity, compassion and respect;’ ^49^ others are closer to ‘taking a biopsychosocial perspective;’ ^10, 15^ others seem more aligned with ‘explores the patients' main reason for the visit, concerns, and need for information;’ ^44^ ‘working with patient’s beliefs and values;’ ^46^ or even ‘emotional support and alleviation of fear and anxiety.’ ^3, 43^

The variation in how different frameworks and definitions of person-centred care carve up the concept suggests that the dimensions they articulate do not pick out clearly distinct and stable entities, but rather identify features in a more continuous conceptual space. This need not imply that the different dimensions ascribed to person-centred care do not capture meaningfully different aspects of it. Rather, it suggests that the dimensions are not easily prised apart from one another.

### iv.  Process and achievement

The framework developed by McCormack and McCance disambiguates person-centred processes and outcomes ^46^. It characterises five core ‘person-centred processes,’ but also identifies a number of supporting factors, including attributes of healthcare professionals and institutions, as well as expected outcomes when those person-centred processes and supporting factors are combined. This suggests that person-centred care is neither just a kind of process nor the end state of a process, but a whole care system, incorporating inputs, processes and outcomes. This highlights an ambiguity that is present in the other frameworks: it is unclear whether person-centred care is best understood in terms of processes or outcomes. That is, is it a set of practices performed with particular intentions—some actions that healthcare practitioners perform—or does it pick out healthcare encounters which in fact achieve some defined standard—some phenomena which are experienced by or happen to patients? These may seem like subtle differences, but in practice they necessitate different approaches to implementation, measurement and assessment—they will influence, for example, who or what investigators take as the object of inquiry and which methods are best suited to ascertaining whether care is indeed person-centred.

## Valuing both determinacy and vagueness

On the one hand, these definitions of person-centred care, taken individually, present it as a relatively clearly defined and demarcated concept with specified components. On the other hand, looked at collectively, the scope, meaning, and constituents of person-centred care become increasingly indeterminate. In this section we argue that this combination of relative determinacy and indeterminacy should not be seen as contradictory, or as constituting a major problem, but as something that should be embraced as important to understanding person-centred care. We suggest that both determinacy and vagueness can have value depending on the purposes for which we are invoking the concept of person-centred care.

The value of determinacy is probably more obvious. Policy makers, professional leaders, educators or patient advocates often wish to encourage and guide institutions and colleagues so they are in a position to offer or support more person-centred care; in such cases it is not sufficient to leave the concept completely unspecified. As suggested in the previous section, what is being called for is not a simple, easily summarised and grasped idea but something multi-dimensional that must be interpreted to inform practice. In this context definitions can provide a useful guide. This is especially important when a shared understanding of what is being pursued is needed to help coordinate caring practices and services (something itself closely allied to models of person-centred care). Without a degree of specification there is also a risk that person-centred care dissolves into mere sloganizing, without any real content, let alone agreed content. Carefully constructed accounts and models of person-centred care—such as the definitional frameworks set out in Table 1—serve to give public substance to the concept.

In other words, the general delineating function of definitional frameworks helps to practically operationalise the concept of person-centred care. Definitions can also be used to play a technical role and can be subject to operationalisation in a more technical sense. For example, an institution may wish to assess whether a particular set of services have become more person-centred; to serve this end they will look to produce a technically specified definition of person-centred care, including a set of indicators that can be used to measure the relevant changes. We can imagine, for example, someone selecting one of the frameworks in Table 1, choosing all or some of the listed dimensions, and for each of the chosen dimensions identifying some empirically observable practices or characteristics of care and experience that can be used as relevant indicators. This process will yield a technical definition that can be applied in a relevant audit, monitoring, or research practice. Quality improvement researchers, for example, may wish to ensure they do not overlook person-centred care in studies of comparative effectiveness.

It is worth paying attention to what is entailed by shifting from a broad definitional framework to a technical definition. It involves a move towards a more precise definition and one that has higher utility for a particular purpose (in this example a specific measure of quality improvement). However, it seems unlikely that anyone would say, without significant qualification, that the technical definition is *better* or somehow gets closer to what person-centred care actually means. Technical operationalisation stipulates what is meant for certain purposes but it is not designed for use outside these purposes. Rather it is designed to narrow down the concept so as to simplify its use. To this end, such technical definitions may, justifiably, place less emphasis on, or even omit, some aspects of person-centred care. For example, the presence of ‘shared decision-making’—widely seen as an important aspect of person-centred care—might be indicated by questions or observations that pick up whether patients are invited to offer their perspectives on, and involved in dialogue about, condition management. These things are useful clues as to the presence of something like shared decision-making, but no-one would think they capture it completely—advocates of shared decision-making are likely to think that it involves, or at least *can* involve, something significantly richer than these basic indicators reveal. In short, technical operationalisation entails a gain in precision or relative determinacy but does so at the expense of a richer, but more indeterminate, sense of the concept. It seems important for a researcher in this instance to be able to operate with both the more and the less highly specified ideas of shared-decision-making, and to be ready to move back and forth between them—for example, in order to recognise the limitations of their definition and the possibility of misinterpretation. This is especially important given the possibility of technical indicators of shared decision-making failing to reflect decision-making that is meaningfully shared, or other key concerns of person-centredness. A healthcare professional may fulfil a set of measured requirements of shared decision-making, for example by using decision aids and making space for discussion of patient needs and values, but might also subtly belittle or coerce their patients.

We want to argue that something closely analogous to this applies at the global level of characterising and thinking about person-centred care. That is, providing definitional frameworks and technical definitions can be useful when we are trying to operationalise a ‘vague’ concept like person-centred care but that does not mean that such definitions are inherently better nor that vagueness is inherently problematic. A level of indeterminacy, and not only the relative determinacy given in specific accounts, can be valuable.

To clarify and justify this claim we return to the characterisation of person-centred care in contrast to healthcare seen as primarily biomedical. In this very general characterisation, the concept of person-centred care does not have very much by way of definite, positive content. It operates something like an invitation to stand back from biomedical thinking—itself a set of attitudes and practices with varied manifestation—and ask what differences should it make if we bear in mind that healthcare involves ‘persons’ and not just ‘objects’ or ‘states’ of one sort or another: are there potentially important attitudes, practices, and purposes that might otherwise risk being relatively neglected? How can we achieve an appropriate integration of, or balance between, biomedical and ‘person-related’ considerations in care? Here what is being indicated by the concept is not a completely empty space but is a large space of conceptual and practical possibilities. The relative open-endedness that attaches to what belongs in the space is a product of the considerable richness both of the idea of persons and the broad scope of what might be meant by centring healthcare around persons and not just biomedicine.

We can illustrate the value of this kind of indeterminacy using an analogy that refers to a single person. Imagine you are organising a party for your friend Janet and you receive the advice—“Just remember you are organising this for Janet.” The advice does not have very much by way of content—we can imagine the adviser does not know anything about Janet—but neither is it empty of content or useless. It invites you to think of the differences the fact that it is for Janet might make. It could be that you know she would be insistent (or less so) in making, or being consulted on, certain kinds of decisions; it could be some of her views, preferences, perhaps along with some more basic facts about her biography, life circumstances, or capabilities seem salient; similarly it might be that you judge that you need to take into account the groups of people and communities that she identifies with or is embedded in. Which of these kinds of things seem to matter most, in what combinations, and what their joint implications are will vary substantially depending upon the person, your relationship with them, and the nature of the event you are organising. Even in a single instance there would likely be competing accounts of what good judgements might look like. The advice itself does not distil into a stable set of clear and distinct dimensions, even if it is possible to think about different aspects of it.

Something like this also applies to a general injunction to treat patients as persons. Such statements are open-ended without being empty and this provides a measure of interpretive elasticity which is crucially important if we want to be able to follow the injunction wisely and adapt it to different circumstances. To issue a reminder that healthcare involves ‘persons’ is to flag up something complex and profoundly important. This is both because persons are multi-faceted and because reference to persons has particular ethical significance. These features give rise to person-centred care being seen as both instrumentally valuable and valuable for its own sake. The fact that persons typically have agency (to some degree), specific points of view, histories and futures, social identities, and contexts, including variable resources, means that diverse ‘person-related’ factors need to be taken into account if healthcare is to ‘work’—to be effective and appropriate. At the same time, ‘persons’ are usually treated as the central focus of ethical reasoning and evaluation such that—whilst accepting that there are different theories about what properly constitutes ethical reasoning—the dignity and well-being of persons, including valuing what matters to them, is seen as ethically fundamental.

In other words the injunction to treat patients as persons points to something of importance but not to something clear-cut. What it means to respond to patients as persons in practice will inevitably require responsiveness to a variety of specific contextual factors—not just the rich and dynamic cluster of needs, capacities, values, and interests that characterise the patient themselves, but also features of the healthcare professionals and institutions in question. There are potentially many different ways of characterising and emphasising these characteristics and relationships and, moreover, different interpretations of what is ethically required or desirable in attending and responding to them. Different people can be committed to ensuring that healthcare successfully integrates or balances biomedical and ‘person-related’ agendas but also come to different conclusions about what this entails both in general and particular cases. These can encompass conclusions which more or less preserve or radically reorient the norms and expectations of both professionals and patients.

Some people may well resist the idea that vagueness and open-endedness can be an advantage. Indeed, some question whether this might disqualify it from being a ‘usable’ concept at all. Gottlob Frege, in his *Foundations of Arithmetic*, argues that all concepts must have sharp boundaries:

To a concept without sharp boundary there would correspond an area that had not a sharp boundary-line all round, but in places just vaguely faded away into the background. This would not really be an area at all; and likewise a concept that is not sharply defined is wrongly termed a concept ^51^.

In order for person-centred care and its components to be concepts at all, according to this account, clear conceptual boundaries must be defined and demarcated. Without clear boundaries, quasi-concepts do not have a sense of their own, and at best acquire a sense only in particular contexts ^52^. This general philosophical concern may be seen to correspond with calls by some healthcare practitioners or researchers for a clear definition of person-centred care—without a clear definition, it might be thought, we don’t really understand what person-centred care means. If accepted, this thought might suggest that we need to discard all or some of the frameworks of person-centred care discussed above and identify the single true or single best definition. Or else it might entail that insofar as the frameworks each pick out a meaningful concept it is not, in most cases at least, person-centred care, but some different, narrower concept.

However, the idea that concepts must have clear boundaries does not accord with the way that many concepts are used and understood. Many ordinary concepts have vague extension and some concepts, in addition, are not clearly boundaried because their definition is contested. The concept of person-centred care arguably shares both these characteristics. In the first category are concepts like ‘bald’—and other ordinary concepts such as ‘heap,’ ‘tall,’ ‘old,’ ‘red’—that do not have sharp boundaries. This doesn’t make them unusable concepts, though it may make them difficult or inappropriate to use for the purposes of categorisation or measurement in some contexts where specificity is needed. While a specific and measurable definition of ‘bald’ or ‘tall’ can be invoked for a particular purpose, it need not apply beyond that and is likely to play a largely pragmatic role rather than make any claims to settle the meaning of the term once and for all. An example of a concept that is relatively indeterminate because its meaning is contested is ‘art’. The concept of ‘art’ has been given many different putative definitions—art is a kind of representation; art is a kind of expression; art is what gives us, or intends to give us, aesthetic experience; art is what you find in a gallery; art is what some group of people take to be art; and so on. Each of these definitions gives a different extension—different entities are included under and excluded from the concept ‘art’ depending on which definition we adopt. But the lack of a settled definition at a theoretical level does not prevent the concept from being understood and from being meaningfully used in ordinary speech, institutional contexts, and scholarly practice. We are arguing the same is true for person-centred care.

Those developing and using more definite definitions and frameworks of person-centred care do not necessarily intend for them to replace other definitions or to be universally applicable. Some frameworks are developed for particular contexts, such as McCormack and McCance’s framework for person-centred nursing and the analysis of person-centredness in relation to rehabilitation developed by Leplege *et al*. ^15, 46^. Other studies suggest that different professional groups tend to emphasise particular aspects of person-centred care, despite some broadly shared conceptual themes ^42^. Some scholars see person-centred care as requiring the adoption of different theoretical perspectives depending on the context and nature of the aspects of person-centred care under consideration; ^47^ others argue that it is central to person-centred care that communication and practice be tailored to the needs and desires of the patient and the requirements of the situation ^53, 54^. But even if individual researchers and practitioners do not intend to close down the concept or field of practice, a theoretical landscape which is largely dominated and shaped by itemised definitions of person-centred care can give the impression that it is a concept that must be specified and operationalised in order to be useful or intelligible. We are suggesting that things are the other way round: the intelligibility of such definitions is grounded in their being answerable to a vaguer, but also richer, concept.

## Making space for multiple accounts

One upshot of this picture of person-centred care is that it not only welcomes vagueness, as one part of the story, but also that it tolerates multiple accounts of person-centredness. We acknowledge that this is not without its complications and will be seen by some as problematic, but we argue that this is something that should be embraced. Acceptance of the pluralism about person-centred care can, we suggest, support valuable debate about which interpretations and dimensions of person-centred care are most important in different contexts and cases and thereby allow for its application across a broad spectrum of settings, making it a highly adaptable concept.

Lack of agreement about the meaning of some concepts can itself sometimes signify something ethically important. Some philosophers have characterised a sub-set of concepts as ‘essentially contested,’ as contrasted with ‘accidentally’ or ‘contingently’ contested ^55^. The core suggestion is that some concepts play such a pivotal role in our practical and ethical lives that they constitute the very terms around which ideological disputes take place. The meaning of concepts such as ‘justice’ or ‘democracy’ cannot be settled by crafting a definition because any putative definition can only ever be one turn in an ongoing dialogue. It is, in effect, part of the function of such essentially contested concepts to help provide the ground of disagreement. And disagreement about their meaning and scope is not something which we should hope to settle once and for all, but something which contributes to a rich and nuanced understanding of our most important normative concepts. To return the example of ‘art,’ closing down debate and disagreement about the meaning and boundaries of art once and for all is undesirable. Disagreement about the meaning of art grapples with questions about the kind of beings that we are, how we see ourselves, how and why we express ourselves, how we interact with and represent the world, and how we organise ourselves socially. This does not, however, mean that it is not sometimes both practical and necessary to identify and use a specific definition that is well-suited to a particular set of purposes.

‘Person’ has been analysed as a concept that ‘is by nature, and ought to be, contested,’ ^56^ and it is plausible to see the concept of ‘person-centred care’ as falling into this category too. On this analysis competing and coexistent accounts of person-centred care can be seen not merely as evidence of open-endedness or elasticity but as embodying key ethical disagreements and inquiries about what matters in healthcare and human life more broadly. If person-centred care is essentially contested in this way, then it is not best understood to be merely descriptive—grounded in observations and picking out some specific, identifiable, and relatively stable object—but also normative—reflecting something about how the world ought to be and what kinds of things matter. It is this normative dimension—the various possible readings of the difference that person-centredness *ought* to make—and not only the descriptive complexity and the need for responsiveness to contexts and cases, that underpins a variety of interpretations. Making space for people to interpret the vague and open label ‘person-centred’ and under-specified but generative normative ideas such as ‘treating patients as persons’ for themselves may be important to facilitate recognition and calling out of the problems that are seen to arise from narrow and inflexible applications of more specific definitions and technical operationalisations of person-centredness.

A vague characterisation of person-centred care can, therefore, for practical purposes, sit alongside and be usefully complemented by a number of more determinate accounts (such as the definitional frameworks summarised above). It is a mistake, however, to think that it can be wholly reduced to them, and especially so by any one of them. As with the concept of art we can learn how to use the idea of person-centred care by learning how to recognise and talk about specific examples, and bundles of examples, in a range of different contexts. We do not have to suppose that there is a single ‘essence’ of the concept that can be precisely formulated in a substantive definition and which every example has in common. Ludwig Wittgenstein famously elucidates an analogous point in relation to the concept of ‘game’, suggesting that when we look across many examples, we find a variety commonalities: ‘we see a complicated network of similarities overlapping and criss-crossing: sometimes overall similarities, sometimes similarities of details.’ ^57^ These examples are not intended to exhaust the concept but to help us understand its meaning and scope. On this account we understand the meaning of a concept like ‘person-centred care’ when we pay attention to how it is used, and thereby learn how we ourselves might use it for specific purposes. Of course someone could choose to say that the vague characterisation of person-centred care that we have used—care that is somehow oriented towards ‘persons’ as contrasted with something narrower like diseases or bodies—is the essence of the idea. We would not strongly object to this way of putting things. The idea of ‘persons’ at least signifies something that has ontological complexity or ‘density’ and also, as we have said, invokes important ethical significance. But we are rather stressing that this characterisation is open to an indefinitely large number of interpretations, and establishes very little about the positive content or application of the idea.

Being able to offer examples that illustrate dimensions of person-centred care can help to translate the concept between contexts. Even very simple examples can play a major role in this respect. A powerful instance is the “Hello my name is” campaign initiated by Dr Kate Granger after her own experiences of being disappointed and frustrated by the frequent absence of introductions in her own care experiences ^58^. Here the emphasis is on reducing some of the unnecessary distance that can arise between healthcare encounters and other everyday encounters which involve people, with names, coming together and acknowledging one another. However, clinicians introducing themselves to patients by no means exhausts person-centre care, despite being an excellent example of it. Given the overview of person-centred care in section two we can easily imagine illustrating other relatively common kinds of practices that might support more person-centred care—such as, examples of people being asked about their experiences and views, with listeners being responsive to their priorities. Examples are helpful, just as the definitional frameworks are, in giving shape to the concept. Indeed, the multi-dimensional definitional frameworks reviewed above can be read as working rather like assemblages of *kinds* of examples.

The variability and range of the frameworks and examples indicates the expansiveness of the concept of person-centred care. This expansiveness can help make sense of the way that the concept has evolved over time and can be adapted for different circumstances and purposes. The broad injunction to reframe biomedical thinking by attending to persons helps to explain why the concept of person-centred care emerged and is arguably most widely developed and applied in more interactive and conversation-based areas of healthcare practice, notably primary care consultations but also areas such as dementia- and mental health care. Here, and in many other cases, there is a very narrow gap between care contexts and everyday inter-personal and social life, and therefore biomedical agendas are often conspicuously nested within broader questions about people’s personal and social well-being. However, person-centred care is also increasingly seen as relevant to acute and emergency care contexts ^59, 60^, suggesting that it might also have the conceptual breadth to apply in areas of medicine that are more tightly determined by relatively defined biomedical processes. Person-centred care in emergency and acute settings might involve, for example, designing care spaces so that patients have the opportunity to discuss sensitive issues in privacy, without the risk of being overheard or interrupted ^61^; keeping patients informed and in comfortable settings if they are subjected to long delays ^62^; and respectful communication, such as making eye contact and acknowledging patients’ fears and concerns ^63^.

Both practical and technical operationalisation allow for the concept of person-centred care to be usefully applied to, and adapted for, a broad range of healthcare settings and circumstances. It is possible to design both qualitative and quantitative measures to evaluate progress against various constructions of person-centredness according to the priorities of relevant stakeholders. Different healthcare contexts will tend to highlight particular dimensions of person-centred care. For example, aspects of person-centredness relating to autonomy and informed choice may be important when several distinct avenues present themselves as means of treating or managing a condition—where shared decision-making is needed to narrow down options or perhaps, for example, in determining a date for an elective surgery. On the operating table, on the other hand, there is limited scope for patient choice in how a surgery is conducted. Instead, dignified and respectful treatment might be more important aspects of person-centred care in this context—how a patient’s unconscious body is treated, how they are dressed and undressed, if necessary, how they are referred to by attending clinicians. In relation to long-term care, it is typically important to see the patient’s illness in the context of their own agency and day-to-day life—their existing and potential capacities for self-management and their circumstances and plans. In broad categories there will be similarities between contexts, but which dimensions are emphasised and how these are interpreted can rightly vary between contexts and, of course, from person to person. It is, likewise, both reasonable and necessary for people undertaking measurement to have specific concerns in mind. These concerns will almost certainly vary between contexts and may evolve over the life of a specific service as different considerations emerge as potentially problematic or as relative priorities. It would not just be practically unmanageable to try to measure every possible dimension of person-centred care at once but impossible to do so in a way that reflected the full range of legitimate interpretations of and perspectives on what the concept entails.

Whilst advocating for the importance of pluralism in relation to person-centred care, we are certainly not open to the idea that ‘anything goes’ when it comes to understanding or using the concept. As we have indicated, we see the concept as having wide bounds but not none. Our account does leave the door open for the existence of bad or inappropriate definitions and conceptions of person-centred care—operational definitions of person-centred care can be very limited in their applicability, over-simplify, or miss out significant aspects, for example. Person-centred care also can be misappropriated in practice, for example when it is used to justify a ‘consumer’ oriented conception of patients, which situates them as independent rational choosers in a relatively unconstrained choice context ^64^. Recognising the possibility of a concept being misused and distorted is crucial for seeing it as a real, dynamic phenomenon which is manifested in practices and actions, rather than conceiving of it as an ideal which is rarely, if ever, reached.

We are also very sympathetic to the thought that multiplying accounts can be problematic. There is little to be gained in constructing more and more readings of the concept. Offering further accounts or definitions requires justification on a case by case basis. Indeed, in many instances, it would make sense to look to already existing accounts rather than attempting to construct more. Furthermore, some efforts to ‘harmonise’ existing accounts—or at least to pay attention to the similarities between them and not just the differences—can be a worthwhile project, one which is undertaken by some of the existing models that are based largely on reviews of existing literature ^10^. This might, for example, even include attempts to develop some broad-brush measures to serve as indicators of person-centredness suitable for application across contrasting sectors and settings. But the upshot of our argument is that there is no reason to presume that such an overarching kind of indicator somehow captures the meaning of person-centred care more than an indicator specially developed for a circumscribed context and purpose. These could both generate useful heuristics to perform different jobs.

Accepting multiple competing accounts does, of course, risk problems of communication and may sometimes serve to disguise conflicting interpretations—with parties looking as if they are talking about the same thing when they are not. However, this is a familiar and manageable problem. For the reasons we have given we do not think this problem is best addressed by looking for a universal definition. Nor do we think this is the best route to take for those who wish to rule out what they see as misconceived or ethically suspect interpretations of the concept. Rather it makes sense for interlocuters—as many already do—to explain that, and why, they are using ‘x framework’ or ‘y definition’ of person-centred care. For the same reason there may sometimes be a good case for people choosing another label to highlight the concerns they have in mind. As with definitions we should perhaps welcome diversity and flexibility with labels—sometimes choosing to use expressions such as ‘patient-centred’, ‘relationship-centred,’ or ‘people-centred’ to accentuate specific sets of considerations.

## Conclusion

We began by reporting the concern some have expressed that the lack of a clear definition of person-centred care creates problems for its implementation and for understanding its value. We are happy to accept this concern whilst, at the same time, asserting something that sounds, on the surface, like its opposite. There are undoubtedly good reasons to give the idea of person-centredness some definite shape and to indicate and illustrate what it means in certain contexts. These definitional exercises can help both to critique practice and to steer practice development. In addition, they can provide evaluative frameworks and measures to compare, monitor, and measure healthcare that aspires to be person-centred. But definitional exercises that are undertaken with an insistence on their finality and universal applicability create obstacles for implementing and understanding person-centred care. In the background of attempts to pin down and specify person-centred care is a more open-ended and indeterminate concept which gives meaning to more determinate definitions and vindicates the practice of trying to make medicine more person-centred. Definitions which seek to supersede and eradicate this vagueness may throw the baby out with the bathwater, by unwittingly casting off the very thing that imparts them credence and relevance.

We have suggested that there are benefits to operating with multiple accounts of person-centredness. This includes some accounts tailored for specific uses, including technically operationalised versions, but also, at the same time, leaving a place for the more open-ended and vague sense of the concept. The latter both enables its adaptability and accommodates practical and ethical disagreements about how it should be interpreted and why it matters in different situations. And technical definitions give shape and content to these manifestations, making them practicable and measurable. Central to person-centred care is a critical, questioning, reflective attitude, asking in relation to every patient and in each clinical encounter, whether their care is appropriately context-sensitive—where templates for good practice and assumptions about what good healthcare looks like cannot be taken to provide conclusive or appropriate answers. This inquiry situates person-centred care in relation to biomedical approaches and evidence-based medicine—not rejecting these important ways of thinking, but rather recognising and exploring their limits as valuable approaches to healthcare practice. Maintaining both vagueness and variety in person-centred care is an essential part of its continuing to embody this reflective attitude, because it enables and upholds its responsiveness to particular people and circumstances. To adopt a fixed definition is to pre-judge in some sense what is required by particular patients and healthcare contexts, which is liable to impede the enactment and achievement of person-centred care.

If person-centredness is vague and varied in the ways we have suggested, its generalisability will be limited—it will not always be possible to validly compare the person-centeredness of different healthcare services or encounters, or to easily export or scale person-centred practices from one context to another. Pragmatic use of specific definitions of person-centredness will mitigate this somewhat by enabling measurement and comparison in constrained contexts. But broadly we see its limited generalisability as a positive feature, not a bug. For being able to compare and scale person-centred care is only valuable if what is measured and implemented is meaningfully person-centred. Any features that are so general as to apply in all instances of person-centred care are liable to be so empty of specific content as to be relatively unhelpful for understanding or implementing it. Making assessments of person-centredness that are not platitudinous means attending to specific features of individuals, healthcare settings and medical practice, and the value of generalisability will be relatively limited in such cases. Of course, a tendency towards non-generalisability puts person-centred care somewhat in tension with clinical guideline-based approaches to evidence-based medicine and healthcare improvement—but this shouldn’t be surprising. Person-centred care entails a radical shift towards individuals which may not be consistent with approaches that prioritise clinical effectiveness and biomedical outcomes. Insofar as person-centred care is promoted and pursued, uncomfortable and costly compromises and trade-offs may be necessary. Though this is not often recognised in the policy documents which largely commend person-centred care as an unqualified good, it is important to recognise the extent to which the concept of person-centredness frames medicine as a social and normative, as much as scientific, practice. Person-centred care challenges us to think again about what medicine is for and, on more radical interpretations, can recentre and destabilise established medical practices and frameworks.

## Data availability

No data are associated with this article.

## Footnotes

^1^Relatedly, person-centred care is also contrasted with service provision that is organised for the convenience of the providers rather than the users of the service ^25^.

^2^There remain further questions, not determined by the constitutive relationship, such as whether person-centred care must be characterised by all of its dimensions, or whether care which fulfils some but not all dimensions is also person-centred; and whether person-centredness admits of degrees. We do not address these issues here.
