# Supplementary material for: Vagueness and variety in person-centred care
Source: Wellcome Open Res. 2022 Jun 16;7:170. [Version 1] doi: 10.12688/wellcomeopenres.17970.1 (PMC9277200; doi:10.12688/wellcomeopenres.17970.1)
Supplement: Peer review history

# Peer review: Reviewer response for version 1

**DOI:** 10.21956/wellcomeopenres.19916.r51110

**Published:** 2022-07-12

**Authors:** Mary Catherine Beach

**Affiliations:** 1 Johns Hopkins Berman Institute of Bioethics, Baltimore, MD, USA

I congratulate Mitchell and colleagues for this satisfying analysis that defends the value of allowing apparent vagueness when operating with multiple accounts of person-centered care rather than seeing this as a conceptual weakness. I found myself nodding in enthusiastic agreement at many of their points, and I have little to add beyond my admiration. Still, I will make a few observations here:

- Advocates for a person-centered approach to healthcare are often called upon to substantiate their position by narrowly defining the concept and proving that this approach will make a difference using the same set of scholarly methods developed by the establishment who rejected it in the first place. It’s a rigged game. Mitchell *et al.* provide a different way to think about this (and other) issue(s), and a helpful vocabulary and set of analogies to clear a path out of the trap.
- Next, it is absolutely true, as the authors point out, that person-centered care has been defined by what it is not. I have often thought that definitions of person-centered care are also defined by what is otherwise ignored in the field of medicine, which has led to a mixing pot of seemingly unrelated components (e.g. attending to patients’ physical comfort and acknowledging the ‘doctor as person’).
- Finally, although Mitchell *et al. *argue that extant definitions of person-centeredness do not adhere to a clearly circumscribed and consistent underlying concept, I tend to think that the general definition offered (“healthcare practice that recognizes, emphasizes, and treats the patient as a *person” *etc.) ***is*** a consistent underlying concept. What accounts for the apparent inconsistencies are that the specification of that concept will differ dramatically in different contexts, but it seems to me that the foundation itself is conceptually clear.

Thank you so much for the opportunity to comment.

Is the work clearly and accurately presented and does it cite the current literature?

Yes

If applicable, is the statistical analysis and its interpretation appropriate?

Not applicable

Are all the source data underlying the results available to ensure full reproducibility?

No source data required

Is the study design appropriate and is the work technically sound?

Yes

Are the conclusions drawn adequately supported by the results?

Yes

Are sufficient details of methods and analysis provided to allow replication by others?

Yes

Reviewer Expertise:

respect; patient-clinician communication; health equity

I confirm that I have read this submission and believe that I have an appropriate level of expertise to confirm that it is of an acceptable scientific standard.

# Peer review: Reviewer response for version 1

**DOI:** 10.21956/wellcomeopenres.19916.r51113

**Published:** 2022-07-04

**Authors:** Lars Sandman

**Affiliations:** 1 National Centre for Priorities in Health, Department of Health, Medicine and Caring Sciences, Linköping University, Linköping, Sweden

Thank you for the opportunity to review the well-written article on *Vagueness and variety in person-centred care* by Mitchell *et al*. I must admit that I am highly partial to the perspective and conclusions in the article – i.e. that the vagueness and variety in definitions of person centred care is not necessarily a problem – but rather a strength in being able to handle a variety of complex situations in healthcare. Personally, I do resist “definitional fascism”, trying to impose monolithic definitions on complex phenomena on people and even more so, attempts to solve complex normative issues through definitions. For example, trying to delineate what healthcare should do, by trying to define the concept of health. In my philosophical training, whether a definition is suitable or not or better or not, should be related to the purpose for what it should be used for. Basically, I think that is what the authors are arguing in defense of, even if not in so many words.

 In the article, the authors show how different dominating conceptualizations express different, but also to some extent, conflicting concepts on person-centred care. They also, in my view convincingly, argue that different situations in a complex healthcare system, need different concepts on person-centred care. Even admitting this, I do have some aspects that I would like to comment upon.

 The authors use the term indeterminacy and claim that there is a value in indeterminacy of a concept. To some extent that might be true – indeterminacy might enable or allow some leeway in interpreting and acting upon a concept. But referring to the example on page 10 with Janet and her party, I am not sure it is the indeterminacy that is valuable there. Rather, what the example shows, is that the statement “Just remember you are organising this for Janet.” is *determinate enough for its purpose* given the context. I would argue that the value of indeterminacy is a different value, than the value in a definition being determinate enough. I think the text might benefit from making that distinction clearer.

 A problem with the vague and multi-faceted account of person-centred care I find the author could address somewhat more in detail is the ‘ideological’ or ‘manipulative’ use such concepts allows. Person-centred care is obviously a ‘thick’ concept, with both descriptive and normative content. Its normative content is, at least nowadays, highly positive. No one, in their right mind, would probably argue that healthcare should be made less person-centred, or person- *un*centred or something of sorts. So, people will generally be accepting that healthcare should be person-centred, and still, we might fill this concept with different (perhaps less acceptable) views on what this actually entails. This, in turn, might be a way to avoid or dodge criticism. I have, on several occasions, in meeting proponents of person-centred care, raised potential normative problems – being faced with the comment ‘But that is not what we mean by person-centredness’ as a way to dismantle the criticism without actually addressing it. This ‘ideological’ stance might become especially problematic when turning person-centredness into policy, competing over scarce resources – and motivating redistribution of resources with vague or multiple concepts of person-centredness which makes it possible to support different and conflicting claims for resources. At the extreme, such a concept might simply be used to benefit the idiosyncratic preferences of strong groups, without much attention to the opportunity cost (perhaps in terms of less person-centredness on some accounts) in other parts of the system or for other groups. Even if the authors touch upon this in the text, I find that they focus more on the clinical level of care – where there is more of an advantage than disadvantage to have a vague and multifaceted concept of person-centredness. I think the text could benefit from addressing the policy, resource-distribution level where the challenges (and benefits) might be somewhat different.

 However, all in all, these comments does not imply a substantial criticism of the analysis and argument in the text – but should rather be seen as a way to complement an already interesting and fruitful perspective on person-centred care.

Is the work clearly and accurately presented and does it cite the current literature?

Yes

If applicable, is the statistical analysis and its interpretation appropriate?

Not applicable

Are all the source data underlying the results available to ensure full reproducibility?

No source data required

Is the study design appropriate and is the work technically sound?

Yes

Are the conclusions drawn adequately supported by the results?

Yes

Are sufficient details of methods and analysis provided to allow replication by others?

Yes

Reviewer Expertise:

I am a professor in healthcare ethics, specializing in priority setting ethics having previously done work on shared decision-making and person centered care.

I confirm that I have read this submission and believe that I have an appropriate level of expertise to confirm that it is of an acceptable scientific standard.
